# Bibliometric analysis of 100 top cited articles of heart failure–associated diseases in combination with machine learning

**DOI:** 10.3389/fcvm.2023.1158509

**Published:** 2023-05-25

**Authors:** Xuyuan Kuang, Zihao Zhong, Wei Liang, Suzhen Huang, Renji Luo, Hui Luo, Yongheng Li

**Affiliations:** ^1^ Department of Hyperbaric Oxygen, Xiangya Hospital, Changsha, China; ^2^ National Research Center of Geriatic Diseases (Xiangya Hospital), Changsha, China; ^3^ Changsha Social Laboratory of Artificial Intelligence, Hunan University of Technology and Business, Changsha, China; ^4^ The Big Data Institute, Central South University, Changsha, China; ^5^ Department of Anesthesiology, Xiangya Hospital, Changsha, China

**Keywords:** machine learning, heart failure, bibliometric analysis, VOSviewer, artificial intelligence, heart diseases

## Abstract

**Objective:**

The aim of this paper is to analyze the application of machine learning in heart failure-associated diseases using bibliometric methods and to provide a dynamic and longitudinal bibliometric analysis of heart failure–related machine learning publications.

**Materials and methods:**

Web of Science was screened to gather the articles for the study. Based on bibliometric indicators, a search strategy was developed to screen the title for eligibility. Intuitive data analysis was employed to analyze the top-100 cited articles and VOSViewer was used to analyze the relevance and impact of all articles. The two analysis methods were then compared to get conclusions.

**Results:**

The search identified 3,312 articles. In the end, 2,392 papers were included in the study, which were published between 1985 and 2023. All articles were analyzed using VOSViewer. Key points of the analysis included the co-authorship map of authors, countries and organizations, the citation map of journal and documents and a visualization of keyword co-occurrence analysis. Among these 100 top-cited papers, with a mean of 122.9 citations, the most-cited article had 1,189, and the least cited article had 47. Harvard University and the University of California topped the list among all institutes with 10 papers each. More than one-ninth of the authors of these 100 top-cited papers wrote three or more articles. The 100 articles came from 49 journals. The articles were divided into seven areas according to the type of machine learning approach employed: Support Vector Machines, Convolutional Neural Networks, Logistic Regression, Recurrent Neural Networks, Random Forest, Naive Bayes, and Decision Tree. Support Vector Machines were the most popular method.

**Conclusions:**

This analysis provides a comprehensive overview of the artificial intelligence (AI)-related research conducted in the field of heart failure, which helps healthcare institutions and researchers better understand the prospects of AI in heart failure and formulate more scientific and effective research plans. In addition, our bibliometric evaluation can assist healthcare institutions and researchers in determining the advantages, sustainability, risks, and potential impacts of AI technology in heart failure.

## 1. Introduction

The explosive growth in the research literature production has led to the need for new approaches to structure knowledge (1). Citations in the medicinal field can reflect the impact of an article in its field, which show that the number of citations is directly related to its worth (2). Bibliometric analysis provides the opportunity to gain an informative understanding of the field of study and promotes interdisciplinary collaboration (3). Bibliometrics is a measurable approach to informatics that analyzes emerging trends and knowledge structures in a field to obtain quantifiable, repeatable, and objective data (4). Top-cited publications in medical journals are also crucial in educating and advancing the next generation of technology (5).

There is a lack of literature on the top heart failure (HF)–related articles, despite the fact that the study of heart disease in recent decades has remained a very popular research topic (6). The epidemiology, pathophysiology, and development of heart failure are complex, and as a result, it can be difficult to determine its origin as well as its diagnosis, prognosis, and course of treatment (7). Artificial Intelligence (AI) is a technology that enables computer systems to simulate, understand, and execute tasks similar to human intelligence. The goal of AI is to create a machine that can think, learn, and adapt to new environments autonomously, handling complex problems and making accurate decisions like a human. Machine learning (ML) has recently been applied in heart failure treatment and it has contributed to the diagnosis, categorization, and prediction of the disease (8).

The aim of the present paper is to propose certain machine learning concepts and provide advice for cardiologists with no machine learning background to participate in the integration of machine learning and medicine and help them conduct research in this area. This paper is also an essential resource for people who are less familiar with the field but are interested in machine learning applications in the field of heart failure.

These excellent articles (9–14) are a good introduction to AI and machine learning–related technologies.

In addition, we provide some key machine learning concepts for busy clinicians.

### 1.1. Traditional rule-based algorithms apply rules to data, while machine learning algorithms learn patterns from data

The main difference between machine learning algorithms and traditional algorithms lies in their input and output. Traditional algorithms are based on rules and logical statements written by programmers that require precise definition of input and expected output. For example, a sorting algorithm requires an unordered list as input to generate a sorted list in ascending order as output.

In contrast, machine learning algorithms are based on statistics and data analysis and can automatically learn patterns and rules from data. Machine learning algorithms can handle raw or unclassified data and learn and adapt from it. The input for machine learning algorithms can be large datasets, and the output is typically a prediction or classification. For example, machine learning algorithms can learn natural language processing rules from large amounts of natural language data to perform natural language understanding or generation tasks more accurately (15).

Another difference is that traditional algorithms are usually deterministic, meaning they always produce the same output for a given input. In contrast, machine learning algorithms can be stochastic or probabilistic, meaning they may produce different output results for a given input because they are based on probabilistic models.

Finally, machine learning algorithms typically require a large amount of data to train the model to improve the accuracy of prediction or classification, whereas traditional algorithms typically use less data. There are several types of machine learning algorithms, from decision trees and support vector machines (SVMs), to highly complex, data-hungry algorithms called neural networks. Neural networks are used in deep machine learning (deep learning or DL), and their ability to analyze large amounts of highly complex data—electronic health record (EHR) data, for example, or the collection of pixels that make up medical images—are especially exciting for cardiology applications (16).

### 1.2. ML algorithms can learn patterns from labeled examples: supervised learning

Supervised learning algorithms need to be trained using existing datasets in order to learn regularity and patterns. These datasets are typically from hospitals or research institutions and include medical images, medical records, and experimental data. The accuracy of supervised learning algorithms depends on the quality and quantity of the datasets used, so a large amount of high-quality data is required to train the model (17).

The advantage of supervised learning algorithms is that they have a clear goal: to predict the labels of interest. But the downside of supervised ML algorithms is that their ability to find interesting patterns in the data is also limited by these labels. Training the right data and deciding on the right answer or label is critical to training and requires a lot of work. Similarly, a major challenge in supervised machine learning is the availability of datasets of sufficient size that have properly annotated labels of interest. However, this is also not necessarily accurate. Therefore, proper labeling of datasets requires active management by physicians and often requires consensus from more than one physician.

### 1.3. ML algorithms can learn patterns without labeled examples: unsupervised learning

Unsupervised learning is a machine learning method that uses unlabeled data for training to discover regularity and patterns in the data without first having labeled data. Unlike supervised learning, unsupervised learning does not require predefined inputs and outputs, but instead lets the algorithm learn the structure and features of the data on its own. The main goal of unsupervised learning is to discover hidden structures in data in order to better understand the data and extract useful information.

The advantages of unsupervised learning include the following:

(i) No need to label data: Unlike supervised learning, unsupervised learning does not require labeled data for training, so it is easier to obtain a large amount of unlabeled data.
(ii) Discover hidden structures: Unsupervised learning can help discover hidden structures and patterns in data to provide better data understanding and analysis.
(iii) More comprehensive data analysis: Unsupervised learning can use a variety of algorithms to analyze data from different perspectives, so as to be able to understand the data more comprehensively.
(iv) Applicable to a variety of fields: Unsupervised learning can be applied to a variety of fields, such as image processing, natural language processing, data mining, etc. (11).

### 1.4. A hot branch of the AI field: deep learning

With the development of medical informatization and digital diagnosis, medical monitoring indicators continue to grow, and the amount of data is getting larger and larger; strong data processing capabilities are urgently needed to provide strong support to the medical field. Deep learning, as a hot branch of the AI field, has developed rapidly in speech recognition and computer vision, and its application in the medical field is increasingly used.

The reason why deep learning is suitable for application in the field of medicine is that it can automatically learn and extract hidden patterns and features from data. This automated process can reduce human interference and errors, and improve the accuracy and efficiency of disease diagnosis and treatment.

## 2. Materials and methods

**Figure 1:**
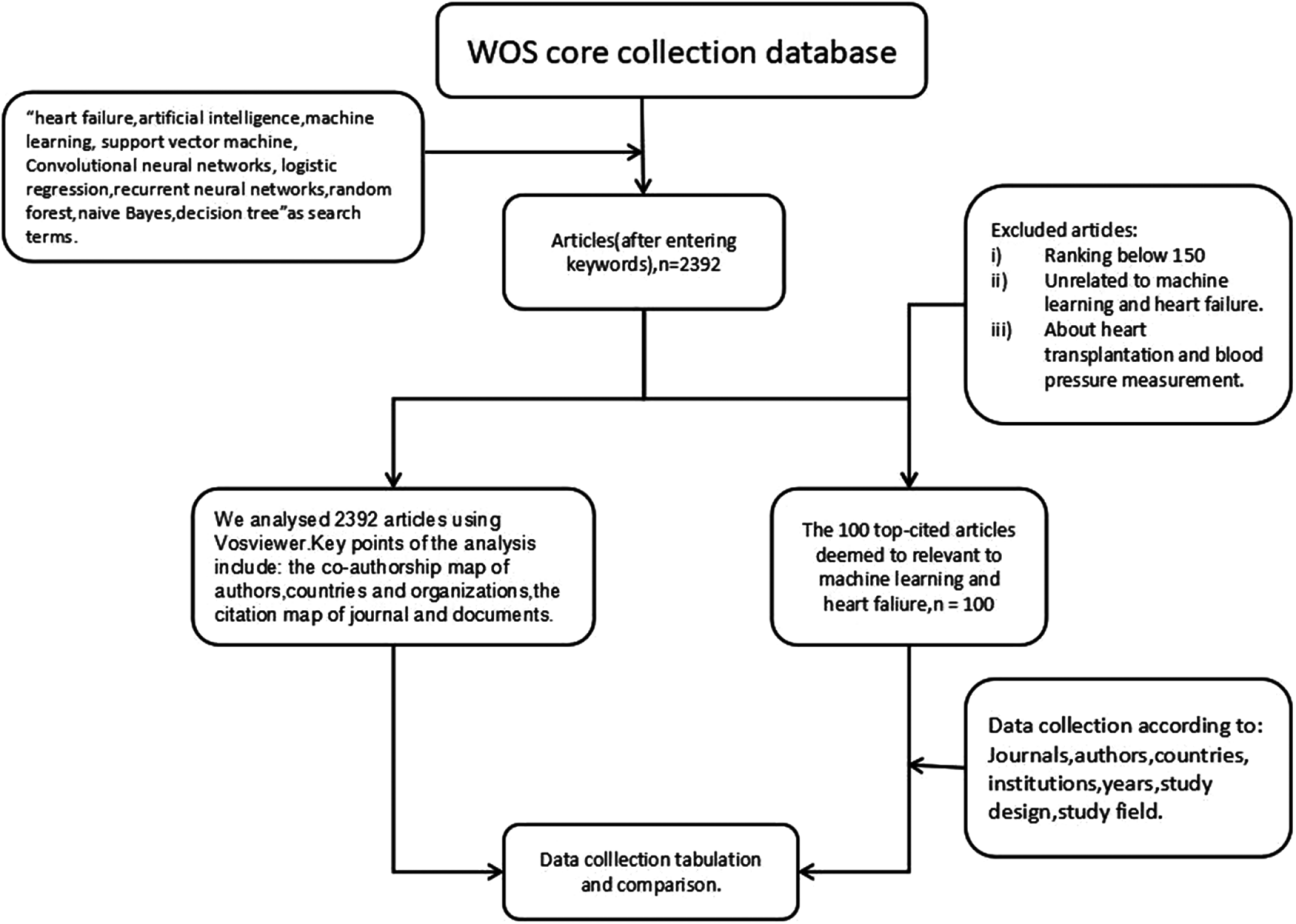
Flow chart showing the methodology used in the study.

Two researchers examined the core collection database Web of Science (WOS) on December 15, 2022. They identified 3,312 articles. In the end, 2,392 papers were included in the study. The papers related to heart failure and machine learning were identified by combing keywords with Boolean operators in the search engine: “heart failure” and “machine learning, AI, Support Vector Machine, Convolutional Neural Networks, Logistic Regression, Recurrent Neural Networks, Random Forest, Naive Bayes, or Decision Tree”. As the search results settled at 2,392 papers, on this basis, we further selected the 100 top-cited articles. Figure 1 shows the selected process. The 100 top-cited articles were compared with the analysis results of all searched articles, and their similarities and differences were analyzed.

The abstract or full text of 100 top-cited articles was read by two independent researchers who manually extracted information about the first and second authors, the journal name, the institute, and other details after evaluating the pertinent articles in accordance with the inclusion and exclusion criteria (18). Exclusion criteria included (i) articles unrelated to heart failure or machine learning and (ii) articles about heart transplantation and blood pressure measurement. Data were extracted from each of the articles by the two researchers and then analyzed by other researchers. The first draft was written by two researchers and all authors commented on the previous editions of the manuscript.

## 3. Results

### 3.1. The basic characteristics of 100 top-cited articles and all articles

In Online Resource 1 (Supplementary Table S1), the final top-100 cited articles were given in order of decreasing number of citations per article. The 2015 article about machine learning in medicine had the most citations, totaling 1,189 (19). The 2019 article about cardiac tissue engineering: state-of-the-art methods and outlook had the fewest citations (47 times) (20).

**Figure 2:**
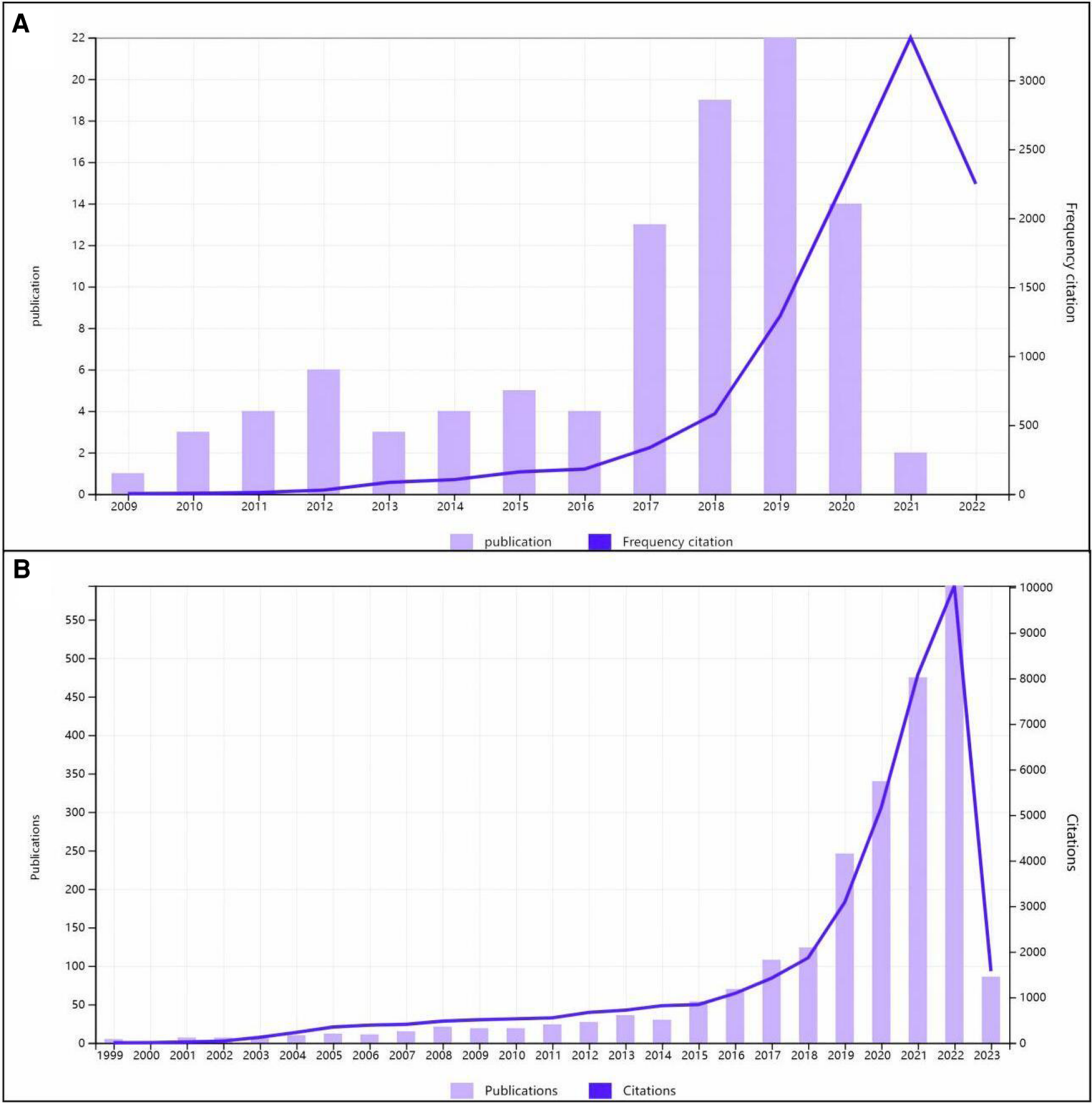
Times cited and publications over time (**A**) with the 100 top-cited articles and (**B**) with the 2,392 articles.

The 100 top-cited articles were all published from 2009 to 2021. The timeline diagram in Figure 2A shows that the period between 2017 and 2022 contains one peak. The majority (68%) of highly cited articles on heart failure were published between 2017 and 2020. All of the articles were published between 1999 and 2023. Figure 2B shows a peak in 2020–2022 and a trend to continue to rise. It can reflect the development history and important nodes in the field of machine learning and heart failure. The growth rate of publications over time was calculated by raising the rate of the number of publications in 2022 over the number of publications in 1999 to the power of 1/23, as shown below. The growth rate is a very important indicator that reflects the development trend in the field. The publication trends of the number of publications each year were also reported (21).

$$\begin{array}{r} \mathrm{Growth}\;\mathrm{rate}=\;((\mathrm{number}\;\mathrm{of}\;\mathrm{publications}\;\mathrm{in}\;\mathrm{the}\;\mathrm{last}\;\mathrm{year}\\ \;÷\mathrm{number}\;\mathrm{of}\;\mathrm{publications}\;\mathrm{in}\;\mathrm{the}\;\\ \;\mathrm{first}\;\mathrm{year}{)}^{1/(\mathrm{last}\;\mathrm{year}-\mathrm{first}\;\mathrm{year})}-1)\times 100 \end{array}$$

### 3.2. Distribution by journal and author

Table 1 lists the journals of the top 100 cited papers in descending order, with the number of articles, average number of citations per publication, and impact factor (2021). In total, 49 journals were included. The following journals had >3 instances: *Artificial Intelligence in Medicine* (*n* = 5), *Journal of the American College of Cardiology* (*n* = 5), *Computer Methods and Programs in Biomedicine* (*n* = 4), *BMC Medical Informatics and Decision Making* (*n* = 3), *European Heart Journal* (*n* = 3), *European Journal of Heart Failure* (*n* = 3), *JACC-Basic to Translational Science* (*n* = 3), and *American Journal of Surgical Pathology* (*n* = 3). A total of 206 authors participated in the preparation of these most-cited articles, and 15 authors participated in the preparation of three or more articles. Daniel Rueckert wrote 5 of the 100 top-cited articles, topping this list. Table 2 includes a list of these 15 authors.

**Table 1:** Journals in which the top 100 cited heart diseases articles were published.

Rank	Journals	Number of articles (%)	Average number of citations per paper	Impact factor^a^ (2021)
1a	*Artificial Intelligence in Medicine*	5	59.2	7.011
1b	*Journal of the American College of Cardiology*	5	199.7	27.203
2	*Computer Methods and Programs in Biomedicine*	4	88	7.027
3a	*BMC Medical Informatics and Decision Making*	3	133.7	3.298
3b	*European Heart Journal*	3	107.3	35.855
3c	*European Journal of Heart Failure*	3	83.7	17.349
3d	*JACC-Basic to Translational Science*	3	50.7	9.531
4a	*Circulation*	2	528.5	39.918
4b	*Circulation-Cardiovascular Imaging*	2	62.5	8.589
4c	*Circulation-Heart Failure*	2	71	10.447
4d	*Computers in Biology and Medicine*	2	50.5	6.698
4e	*Critical Care*	2	70	19.334
4f	*Expert Systems With Applications*	2	70	8.093
4g	*IEEE Access*	2	57.5	3.476
4h	*IEEE Journal of Biomedical and Health Informatics*	2	93	7.021
4i	*IEEE Transactions on Biomedical Engineering*	2	141.5	4.756
4j	*IEEE Transactions on Visualization and Computer Graphics*	2	80	5.226
4k	*International Journal of Medical Informatics*	2	123.5	4.73
4l	*Journal of Biomedical Informatics*	2	63.5	8
4m	*Journal of the American Society of Echocardiography*	2	52.2	7.722

^a^ Journal impact factor is based on Thomson Reuters Web of Knowledge Journal Citation Reports Ranking (2021).

**Table 2:** Authors with three or more top-cited articles.

Rank	Author	Number of articles
1	Ruecker D	5
2a	Acharya UR	4
2b	Johnson KW	4
2c	Sengupta PP	4
2d	Stewart WF	4
3a	Cook SA	3
3b	Dawes TJW	3
3c	De Marvao A	3
3d	Dudley JT	3
3e	Krittanawong C	3
3f	Melillo P	3
3g	Sanchez-martinez S	3
3h	Shameer K	3
3i	Tang WHW	3
3j	Wang Z	3

**Figure 3:**
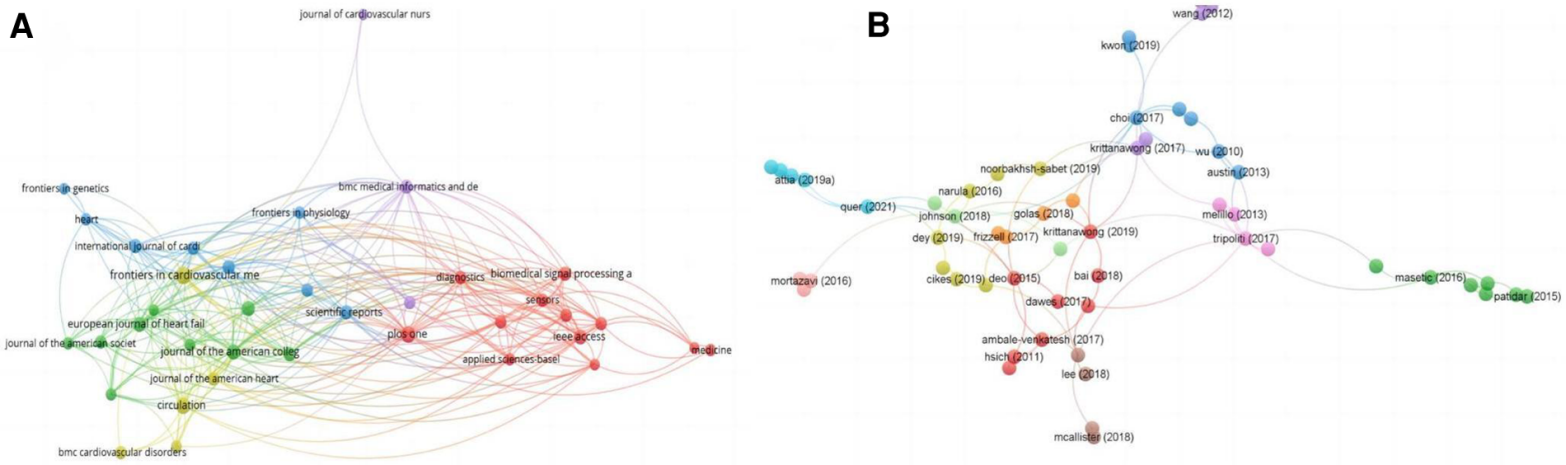
Visualization knowledge maps of citation. (**A**) Citation map of journal; (**B**) citation map of documents. Different color indicates different clusters. The size of the nodes represents the count of citations. The distance between the two nodes indicates their correlation.

**Figure 4:**
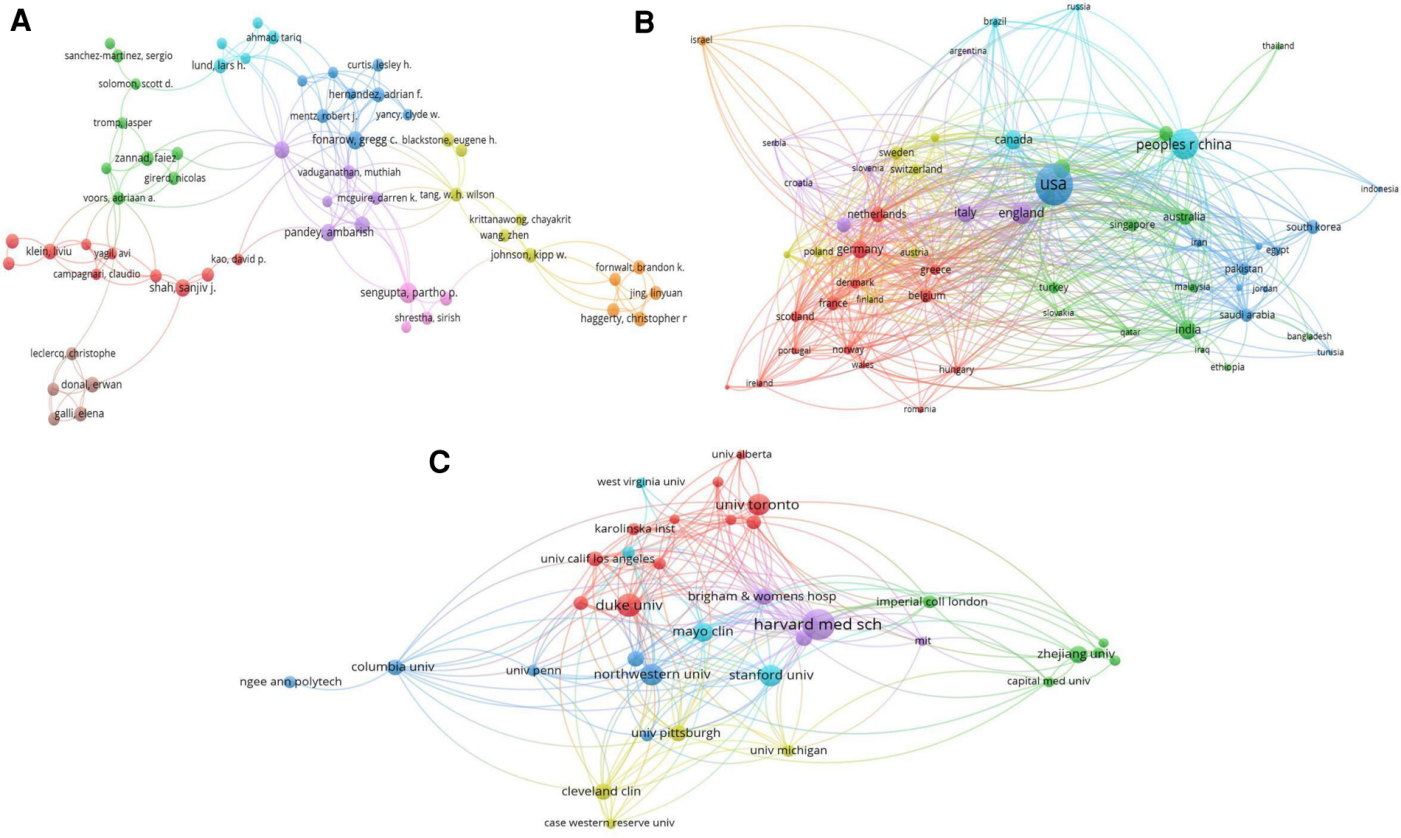
Visualization knowledge maps of the co-authorship. (**A**) Co-authorship map of authors that indicates the authors that cooperate in the field of heart failure. (**B**) Co-authorship map of countries. (**C**) Co-authorship map of organizations. Different colors indicate different clusters and the size of nodes indicates the number of publications. Thickness of the lines represents link strength of the countries.

Analyses of the data were descriptive in nature (22). According to the total link strength (TLS), which measures the overall strength of connections between a certain researcher and the co-authors of other articles, projects were divided into clusters. The citation map of journals and the co-authorship map of authors for the 2,392 articles are shown in Figures 3A, 4A. A total of 43 journals have contributed to machine learning in heart failure. Among them, *Frontiers in Cardiovascular Medicine* was the leading journal with 78 documents, followed by *Circulation* with 58 documents, and *PLoS One* with 52 documents. The top three co-authorship triads of authors were Pandey Ambarish (TLS = 38), Segar Matthew W. (TLS = 36), and Acharya U. Rajendra (TLS = 35). In comparing highly cited articles to all articles, we found a big difference. The impact rankings for each country and each journal provided are based on citation rates (23).

### 3.3. Analysis of high-cited references

The most-cited reference was published in the *New England Journal of Medicine* and authored by Alan S. Maisel in 2002. The second most-cited reference was published in *Circulation* and authored by Rahul C. Deo in 2015. The third most-cited reference was published in the *Cochrane Database of Systematic Reviews* and authored by Jasvinder A. Singh in 2011.

These top three articles were analyzed. The most-cited reference by A. S. Maisel in 2002 (24) reported that measurements of B-type natriuretic peptide added significant independent predictive power to other clinical variables in models predicting which patients had congestive heart failure, using multiple logistic regression analysis. Used in conjunction with other clinical information, rapid measurement of B-type natriuretic peptide is useful in establishing or excluding the diagnosis of congestive heart failure in patients with acute dyspnea.

The second most-cited reference by R. C. Deo in 2015 reported that part of its effort was to identify what obstacles there may be in the change of the practice of medicine through statistical learning approaches, and discuss how these might be overcome (19).

The third most-cited reference by J. A. Singh in 2011 used mixed-effects logistic regression using arm-based random-effects models within the empirical Bayesian framework, and planned to combine the results of biologics used in many conditions to obtain much-needed risk estimates (25). The citation map documents are shown in Figure 3B.

### 3.4. Countries and organizations of top 100 cited articles

Countries and institutes of origin of the top 100 cited articles are listed in Tables 3, 4. A total of 24 different countries contributed these 100 articles. The United States (*n* = 50) with half of the articles was the most prolific country, followed by China (*n* = 7), England (*n* = 5), Singapore (*n* = 4), Canada (*n* = 4), Spain (*n* = 4), Australia (*n* = 3), Turkey (*n* = 3), Italy (*n* = 3), and others. The institutes with the largest number of publications were Harvard University in the United States (*n* = 17) and University of California in the United States (*n* = 10). Icahn School of Medicine at Mount Sinai in Egypt produced eight articles. Two institutes produced seven articles: Mayo Clinic in the United States and Stanford University in the United States. As for all the articles, the co-authorship map of countries and institutes is summarized in Figures 4B,C. The top three countries in co-authorship were the United States, China, and England. The top three institutes in co-authorship were Harvard Medical School in the United States, Duke University in the United States, and University of Toronto in Canada. The field of AI healthcare attracts research from all over the world, but high-income countries are a major force in healthcare-related AI research. The United States alone contributes about half of the research in healthcare-related AI research (26), staying far ahead of other countries in terms of both quantity and quality of its contribution in this area. For researchers, understanding the ranking results can help them better choose suitable research partners, publish research results in appropriate journals, and participate in academic conferences.

**Table 3:** Original countries of the top-cited articles.

Rank	Countries	Number of article
1	United States	50
2	China	7
3	England	5
4a	Singapore	4
4b	Canada	4
4c	Spain	4
5a	Australia	3
5b	Turkey	3
5c	Italy	3

**Table 4:** Original institutions with two or more top-cited articles.

Rank	Institutions	No. of articles
1a	Harvard University, United States	17
1b	University of California System, United States	10
2	Icahn School of Medicine at Mount Sinai, Egypt	8
3b	Mayo Clinic, United States	7
3c	Stanford University, United States	7
4a	Brigham Women's Hospital, United States	6
4b	National Heart Centre Singapore, Singapore	6
5a	Columbia University	5
5b	Imperial College London, United Kingdom	5
5c	National University of Singapore, Singapore	5
5d	Northwestern University, United States	5
5e	University of California Sanfrancisco, United States	5
5f	University of Texas System, United States	5
6a	Cleveland Clinic Foundation, United States	4
6b	Ngee Ann Polytech, Singapore, Singapore	4
6c	Siemens Ag University of Texas, United States	4
6d	Yale University, United States	4
6e	Southwestern Medical Center Dallas, United States	4

### 3.5. Heart diseases and machine learning methods of the top-cited articles

Causes of heart failure included atrial fibrillation (*n* = 8), cardiomyopathy (*n* = 20), cardiovascular disorders (*n* = 22), and coronary artery disease (*n* = 6). Moreover, we discovered that SVM appeared 22 times and SVM utilized in the 100 publications accounted for the majority. Numerous deep learning techniques, including Recurrent Neural Networks, Convolutional Neural Networks, and others, were also utilized. Heart disorders frequently had certain consequences or causative diseases in addition to the link to machine learning, for example, pneumonia (*n* = 2), diabetes (*n* = 5), hypertension (*n* = 5), and stroke (*n* = 5). The 100 studies suggest that machine learning techniques can be used to discover interactions between heart failure and other diseases.

**Figure 5:**
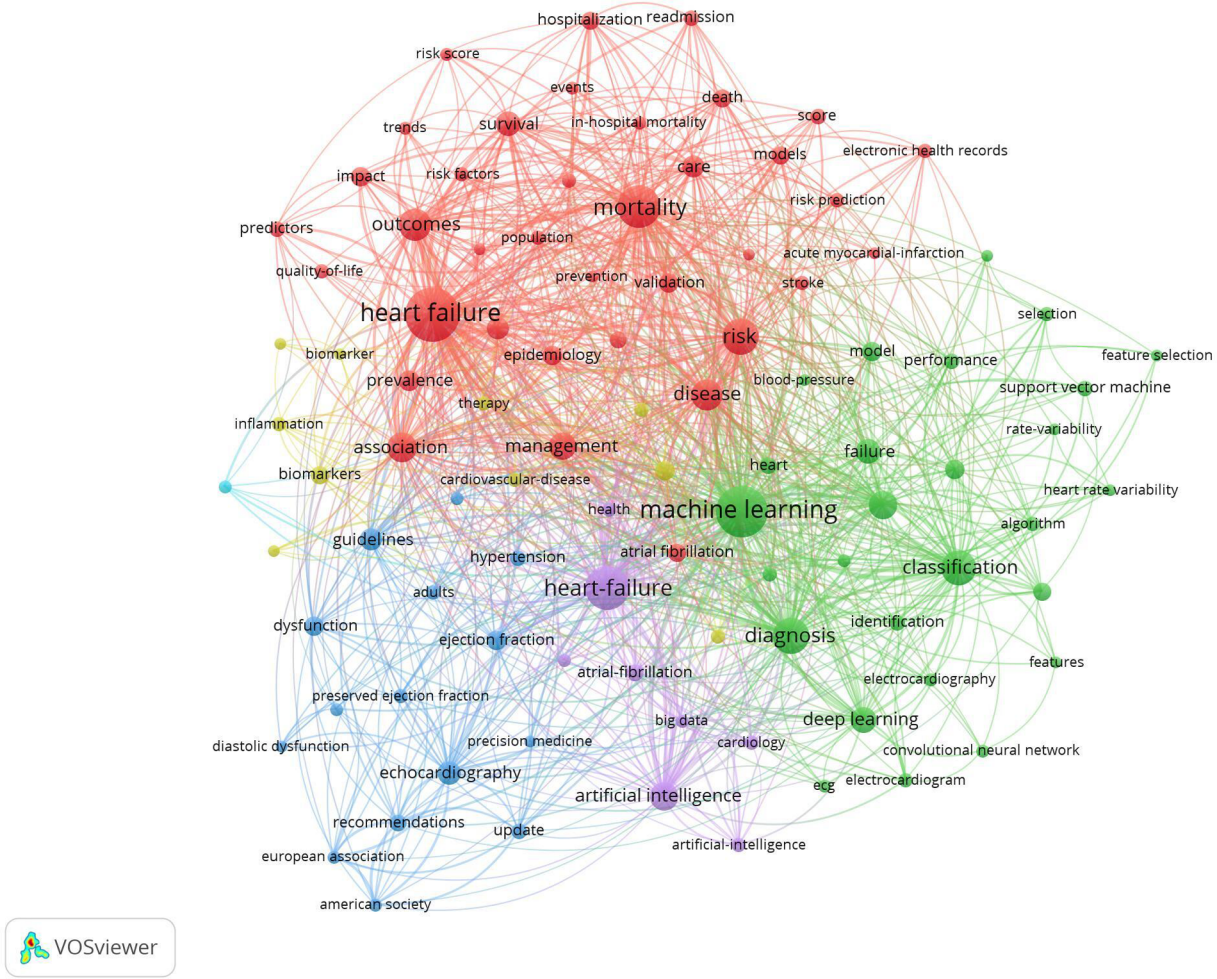
Visualization of keyword co-occurrence analysis. The size of nodes indicates the frequency of occurrences of the keywords. The lines between the nodes represent their co-occurrence in the same publication. Shorter distance between two nodes means higher co-occurrence of two key words.

Figure 5 shows the co-occurrence map of keywords and four research directions. The blue cluster includes heart failure, machine learning, AI, and precision medicine. The red cluster includes risk score, risk prediction, in-hospital mortality, and hospitalization. The green cluster includes prediction, classification, electronic health records, feature selection, and identification. Finally, the yellow cluster includes electrocardiograph, biomarker, and recommendations. AI technology research in recent years has involved more healthcare fields and produced more diverse keywords. It is possible that the higher diversity of keywords of healthcare-related AI research diluted the citation bursts. From the perspective of citation rates, deep learning, SVM, big data, and electronic health records have the greatest impact on heart failure research. We have realized that deep learning has been gradually applied to the medical field, surpassing traditional machine learning methods frequently mentioned in the 100 top-cited articles.

## 4. Discussion

### 4.1. Bibliometrics is of significance in promoting scientific development and progress

Bibliometrics not only demonstrates trends in heart failure research but also shows historical promises for scientific growth (27). Moreover, bibliometric analysis might shed light on the most popular subjects in heart disease (28).

The most citations that the top 100 journal papers from 1988 to 2022 ranged from 47 to 1,189. The collection of publications identifies topics that mirror the development of research on cardiac disorders throughout this 34-year span. Even though it might not be possible to thoroughly evaluate every one of these highly referenced papers, certain findings can be made. The characteristics of these important articles on heart problems are summed up in our current research.

### 4.2. The United States is leading the way in the area of heart failure and machine learning

The United States accounts for 50 of the 100 top-cited articles globally, which confirms the tremendous impact on medical science research in the United States with its large scientific population and the sufficient financial resources which are available to the scientific communities, demonstrating its dominance in the fields of research, technology, and medicine. The United States is a pioneer in many other fields as well (29). The United States hosts many prestigious journals, which may be the reason why publications there account for the vast majority. Nevertheless, researchers prefer to cite papers from their own countries. American researchers may similarly favor papers from their own country (30, 31). For papers published in WOS, this work is with significant per-capita contributions from all industrialized nations as well as work in the majority of developing nations. Global and similarly multi-institutional collaborations in cardiac AI/ML exist within the United States.

It is undeniable that citation analysis within a specific subject may always provide a wealth of information about journals, organizations, and authors that is useful for identifying important publications and high-impact journals. It shows the trajectory of heart failure research as well as a historical perspective on scientific advancement in the subject. Through the analysis of the statistics of the journals, authors, and countries of the first 100 citations, we can learn advanced medical technologies and concepts, learn the advanced nature of the country that plays a leading position in this field, understand the future development trend of the field, and timely improve the shortcomings and shortcomings of the data analysis methods used by medical scientists. Most importantly, our statistical results are intended to provide these healers with the direction and orientation to solve problems (32).

### 4.3. Range of machine learning methods in heart diseases in United States, Europe, and Asia

**Figure 6:**
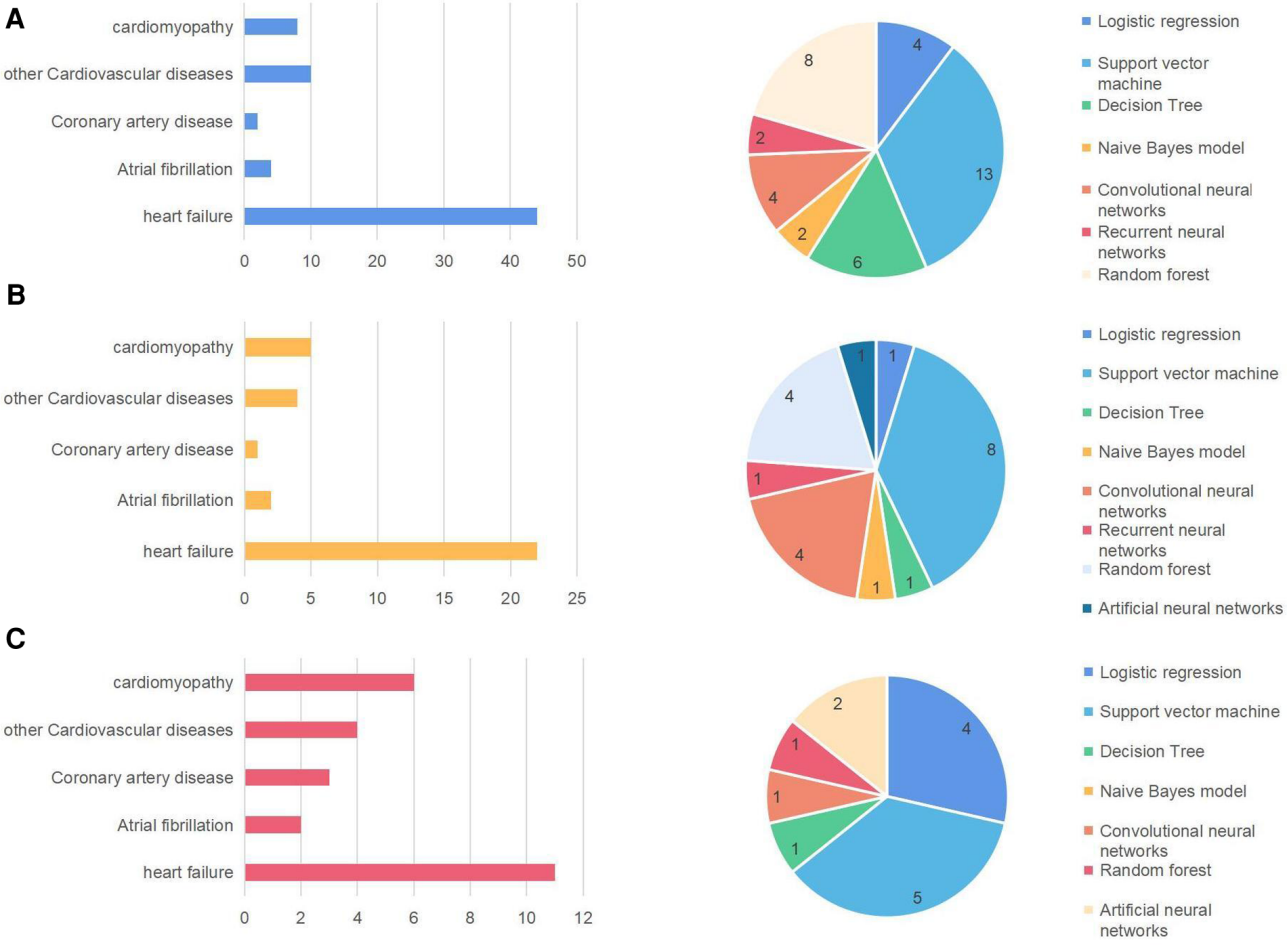
Major heart diseases and machine learning methods in the United States (**A**), Europe (**B**), and Asia (**C**).

We divided the 100 articles based on their original countries for analysis: the United States, Europe, and a few Asian countries (mainly from China). Figure 6 displays the articles from the three areas that examine the main cardiac conditions and machine learning techniques. The tables on the left of the three regions show heart failure and its common causes (33). It demonstrates that the worldwide research results about origins of heart diseases are much the same. SVM, a binary classification method that uses machine learning techniques, occurs most frequently. Its basic model is defined in the feature space of the largest interval linear classifier. Its advantage is that only a small number of support vectors determine the outcome, which not only helps identify important samples but also eliminates redundant samples (34). These techniques have been widely adopted by medical research to predict disease and survival.

However, the interesting thing is that in the article “Comparing different supervised machine learning algorithms for disease prediction,” we found the following description. They discovered that the SVM algorithm is applied most frequently (in 29 studies) followed by the Naïve Bayes algorithm (in 23 studies). However, the Random Forest (RF) algorithm showed superior accuracy comparatively. Of the 17 studies where it was applied, RF showed the highest accuracy in 9 of them, i.e., 53%. This was followed by SVM which topped in 41% of the studies it was considered (35). We also found that different diseases use different machine learning models to achieve different accuracy.

### 4.4. Analysis of the application of machine learning in heart failure

Millions of individuals die from cardiovascular disease yearly worldwide. Heart attack (induced by obstruction of blood vessels), stroke (caused by occlusion or rupture of cerebral blood vessels), and heart failure are the three main heart and blood vessel diseases (caused by the inability of the heart to pump enough blood to the body). Prediction of patients’ status and prognosis based on clinical and laboratory data is crucial since severe heart failure can result in mortality (36).

Using machine learning, heart failure may be predicted, detected, and treated with high reliability and accuracy. Among these, data analysis and model building for heart failure frequently use methods like Neural Networks, Support Vector Machines, Decision Tree, and Random Forests. Physiological data, electrocardiograms, medical records, and images are just a few of the numerous data sources that offer rich data resources and research foundations for application of machine learning in heart failure (37).

Since SVM appears the most, we analyzed the articles in which this keyword appeared. We found that most of the SVM used by these authors are not traditional SVM models. Here are some examples: (i) SVM and boosting algorithms (38); (ii) an expert system that stacks two (SVM) models. The first SVM model is linear and L-1 regularized and the second SVM model is L-2 regularized (39); (iii) RBF kernel-based SVM (40); (iv) a boosted C5.0 tree, as the base classifier, was ensembled with a SVM, as a secondary classifier (41); (v) the proposed prediction SVM model with particle swarm parameter (42). These are some of the representatives of the 100 top-cited articles. It is all known to us that just building a working AI model does not make it usable. We have to combine and compare different models to improve our accuracy, and we have to pay attention to the development of technology and update our technology, because each model has its advantages and disadvantages. For example, random forests are suitable for handling large datasets, and this ensemble-based classifier may outperform a single classifier, while SVM is less prone to overfitting and performs well in classifying semistructured or unstructured data such as text, images, etc. (35). We can learn more about the differences from this paper.

### 4.5. Specific applications of machine learning

As most machine learning algorithms are “data agnostic” (43), the case studies are organized not by clinical data types or disorders, but rather by machine learning use cases.

Machine learning has some of the following key capabilities

Denoising and image enhancement: Machine learning has been used to address the clinical issue of image post-processing and denoising across a variety of modalities. For ultrasound, CT, MRI, and clinical imaging, neural network-based denoising has proven beneficial due to the complicated patterns of noise found in clinical imaging (44). In terms of clinical utility, machine learning has the ability to reduce the time and effort required for image post-processing as well as the discrepancies in data processing between operators, vendors, and institutes (45). Most machine learning denoising techniques now employ supervised learning techniques, where the model is trained to approximately represent proprietary denoising software as a real label (46). A crucial next step to demonstrate its usefulness in clinical practice is the use of machine learning–based image denoising to monitor changes in diagnostic performance. To test image denoising on a broader scale, several clinical trials are now underway (47).

Feature extraction, feature selection, and feature representation: Feature selection is the process of choosing the most pertinent characteristics from a vast number of features, aiming to increase the precision and interpretability of the prediction model. For instance, feature selection techniques based on Decision Trees or Logistic Regression algorithms might be used. Representing important features in a simpler and more unified form is called feature extraction. For example, myocardial wall thickness measurement is a crucial diagnostic, monitoring, and therapeutic tool for the identification of coronary heart disease and cardiomyopathy. The outlines and thickness of the myocardial wall can be automatically extracted and determined using feature extraction methods in medical image analysis (48).

Measuring cardiac flow is vital for evaluating cardiac function and lesions as it provides important diagnostic information about the volume of blood the heart pumps each minute (49). Heart disorders including coronary artery disease and heart failure may be diagnosed and treated using feature extraction algorithms. These algorithms can automatically extract the contours of the ventricles and blood arteries and determine the flow rate and velocity (50).

Deep learning algorithms achieve higher classification performance through automatic extraction of these characteristics through intricate nonlinear combinations of input data (51). Data may be compared between institutes using these extracted features, and by integrating features from various data types, it is possible to create a multimodal image of a specific patient or disease by integrating features from various data types. This study will continue to benefit from concurrent experiments using small datasets as well as synthesizing larger datasets from smaller datasets, because training a model to execute these fundamental tasks frequently requires a lot of data labeling.

With a small amount of data available, synthetic knowledge synthesis (SKS) can be a useful tool for heart failure feature extraction. Existing data can be combined with domain expertise to create more data samples, enlarging the dataset and enhancing the model's performance. Synthetic knowledge synthesis can also aid in avoiding issues such as overfitting. However, SKS cannot deal with all issues. There is just one viable answer and more experimental testing is needed to determine whether it applies to certain datasets. For effectiveness, it is necessary to combine SKS with other machine learning approaches, such as feature selection, classifier selection, and so on (52).

### 4.6. Strength and limitations in our analysis process

#### 4.6.1. Strength

The Web of Science database is an authoritative citation database with a worldwide scope. It includes the core journals with the greatest academic influence across a range of disciplines and the literature can, to a certain extent, reflect cutting-edge global development trends in certain discipline or field. Objective assessment of the current state and level of scientific advancement is possible through observing quantity and quality of the produced scientific materials.

The 100 top-cited articles were compared with all articles retrieved. Readers can find suitable and commonly used methods as well as valuable conclusions from their comparison and analysis. They can also learn development laws from the present paper and realize better combination of medicine and machine learning.

#### 4.6.2. Limitations

Some pertinent articles might have been overlooked due to the keywords used. Second, the citation count could be inflated by self-citations, errors, or insufficient statistics. Because of this, older articles may receive more citations at the time of retrieval and study because they have received more citations over a longer period of time (53). The Web of Science core collection database was used as the literature source. Various databases have significant disparities and if the literature was procured from another database, even more variations would be observed in the citations or articles (54). Most importantly, some keywords, although they were ranked as top keywords, were uninformative by themselves (risk, model, and system) and *could not be analyzed*.

Notwithstanding the potential of machine learning in the study and treatment of heart failure, there are a number of problems and difficulties that must be resolved. For instance, gathering a sufficient amount of high-quality data to train and verify machine learning algorithms can be challenging. On the other hand, heart failure is a complicated disease and involves interaction of multiple tissues and organ systems. Therefore, data integration and model interpretability are the major challenges preventing widespread assimilation into clinical practice (55), which might lead to practical application issues, particularly in clinical decision-making.

## 5. Conclusion

Our analysis also depicted research trends of AI-related health research: (i) the growth rate of heart failure–related ML publications has grown rapidly in the past two decades and the rate showed a trend of continuous growth; (ii) high-income countries are the main force of HF-related AI research; (iii) ML is being increasingly employed in the field of heart failure. Future research trends will most likely include more accurate predictive models, personalized treatment, real-time monitoring, and early warning systems; (iv) although SVM appears the most, what cannot be ignored is the combination of different models. Improving accuracy and reducing complexity is the goal of doctors.

## Data Availability

The original contributions presented in the study are included in the article/Supplementary Materials, further inquiries can be directed to the corresponding authors.
