# Supplementary material for: Bibliometric analysis of 100 top cited articles of heart failure–associated diseases in combination with machine learning
Source: Front Cardiovasc Med. 2023 May 25;10:1158509. doi: 10.3389/fcvm.2023.1158509 (PMC10248156; doi:10.3389/fcvm.2023.1158509)
Supplement: Supplementary file 1 [file Datasheet1.pdf]

Supplementary Material

Bibliometric analysis of 100 top-citied articles of heart failure associated diseases in combination with machine learning

Xuyuan Kuang[1,2†],Zihao Zhong[3†],Wei Liang[3*],Suzhen Huang[4*],Renji Luo[3],Hui Luo[2,5],Yongheng Li[3]

*** Correspondence:**

Wei Liang:weiliang@csu.edu.cn

Suzhen Huang:huangsuzhen@csu.edu.cn

1 Supplementary Tables

Rank	Title	Total Citations	Average per Year
1	Machine Learning in Medicine	1095	136.88
2	An artificial intelligence-enabled ECG algorithm for the identification of patients with atrial fibrillation during sinus rhythm: a retrospective analysis of outcome prediction	385	96.25
3	Artificial Intelligence in Precision Cardiovascular Medicine	377	62.83
4	Artificial Intelligence in Cardiology	371	74.2
5	Using recurrent neural network models for early detection of heart failure onset	359	59.83
6	Automated cardiovascular magnetic resonance image analysis with fully convolutional networks	299	59.8

7	Comparing different supervised machine learning algorithms for disease prediction	262	65.5
8	Cuffless Blood Pressure Estimation Algorithms for Continuous Health-Care Monitoring	247	41.17
9	Cardiovascular Event Prediction by Machine Learning The Multi-Ethnic Study of Atherosclerosis	244	40.67
10	Federated learning of predictive models from federated Electronic Health Records	227	45.4
11	Prediction Modeling Using EHR Data Challenges, Strategies, and a Comparison of Machine Learning Approaches	210	16.15
12	Artificial Intelligence in Cardiovascular Imaging JACC State-of-the-Art Review	204	51
13	Machine-Learning Algorithms to Automate Morphological and Functional Assessments in 2D Echocardiography	195	27.86
14	Clinical applications of machine learning in cardiovascular disease and its relevance to cardiac imaging	179	44.75
15	Titin-truncating variants affect heart function in disease cohorts and the general population	174	29
16	Prediction of 30-Day All-Cause Readmissions in Patients Hospitalized for Heart Failure Comparison of Machine Learning and Other Statistical Approaches	173	28.83
17	Analysis of Machine Learning Techniques for Heart Failure Readmissions	165	23.57
18	Congestive heart failure detection using random forest classifier	162	23.14

19	Using methods from the data-mining and machine-learning literature for disease classification and prediction: a case study examining classification of heart failure subtypes	162	16.2
20	Video-based AI for beat-to-beat assessment of cardiac function	160	53.33
21	Multiscaled Fusion o Deep Convolutional Neural Networks for Screening Atrial Fibrillation From Single Lead Short ECG Recordings	141	28.2
22	A Hybrid Intelligent System Framework for the Prediction of Heart Disease Using Machine Learning Algorithms	127	25.4
23	Automated diagnosis of coronary artery disease using tunable-Q wavelet transform applied on heart rate signals	122	15.25
24	Deep learning for cardiovascular medicine: a practical primer	114	28.5
25	Machine Learning of Three-dimensional Right Ventricular Motion Enables Outcome Prediction in Pulmonary Hypertension: A Cardiac MR Imaging Study	113	18.83
26	Artificial Intelligence Transforms the Future of Health Care	108	27
27	Machine learning-based phenogrouping in heart failure to identify responders to cardiac resynchronization therapy	106	26.5
28	Deep-learning cardiac motion analysis for human survival prediction	104	26
29	RetainVis: Visual Analytics with Interpretable and Interactive Recurrent Neural Networks on Electronic Medical Records	104	26
30	Comparison of Machine Learning Methods With Traditional Models for Use of Administrative Claims With Electronic Medical Records to Predict Heart Failure Outcomes	102	34

31	Machine learning can predict survival of patients with heart failure from serum creatinine and ejection fraction alone	101	33.67
32	Integrated Use of Bedside Lung Ultrasound and Echocardiography in Acute Respiratory Failure A Prospective Observational Study in ICU	100	11.11
33	Machine Learning Methods Improve Prognostication, Identify Clinically Distinct Phenotypes, and Detect Heterogeneity in Response to Therapy in a Large Cohort of Heart Failure Patients	99	19.8
34	Early prediction of circulatory failure in the intensive care unit using machine learning	98	32.67
35	Computer-aided diagnosis of atrial fibrillation based on ECG Signals: A review	97	19.4
36	A machine learning model to predict the risk of 30-day readmissions in patients with heart failure: a retrospective analysis of electronic medical records data	95	19
37	Phenomapping of patients with heart failure with preserved ejection fraction using machine learning-based unsupervised cluster analysis	94	23.5
38	Novel Wearable Seismocardiography and Machine Learning Algorithms Can Assess Clinical Status of Heart Failure Patients	93	18.6
39	Recent Advances in Seismocardiography	88	22
40	Machine Learning Prediction of Mortality and Hospitalization in Heart Failure With Preserved Ejection Fraction	87	29
41	Machine Learning to Predict the Risk of Incident Heart Failure Hospitalization Among Patients With Diabetes: The WATCH-DM Risk Score	83	20.75

42	Heart Failure: Diagnosis, Severity Estimation and Prediction of Adverse Events Through Machine Learning Techniques	82	13.67
43	Machine Learning Analysis of Left Ventricular Function to Characterize Heart Failure With Preserved Ejection Fraction	80	16
44	Development and validation of Risk Equations for Complications Of type 2 Diabetes (RECODe) using individual participant data from randomised trials	78	13
45	Predictive models for hospital readmission risk: A systematic review of methods	77	15.4
46	Adaptive neuro-fuzzy inference system for classification of ECG signals using Lyapunov exponents	76	5.43
47	Machine Learning and the Future of Cardiovascular Care JACC State-of-the-Art Review	75	37.5
48	An integrated framework for finite-element modeling of mitral valve biomechanics from medical images: Application to MitralClip intervention planning	75	6.82
49	Derivation and Validation of Machine Learning Approaches to Predict Acute Kidney Injury after Cardiac Surgery	74	14.8
50	Outlier detection for patient monitoring and alerting	74	7.4
51	Decision tree for adjuvant right ventricular support in patients receiving a left ventricular assist device	74	6.73
52	An Optimized Stacked Support Vector Machines Based Expert System for the Effective Prediction of Heart Failure	72	18
53	Predictive modeling of hospital readmissions using metaheuristics and data mining	72	9

54	Application of support vector machine for prediction of medication adherence in heart failure patients.	72	5.54
55	Clustervision: Visual Supervision of Unsupervised Clustering	71	14.2
56	Electrocardiogram analysis using a combination of statistical, geometric, and nonlinear heart rate variability features	71	5.92
57	Incorporating expert knowledge when learning Bayesian network structure: A medical case study	70	5.83
58	PREDICTIVE MODELING OF HOSPITAL READMISSION RATES USING ELECTRONIC MEDICAL RECORD-WIDE MACHINE LEARNING: A CASE-STUDY USING MOUNT SINAI HEART FAILURE COHORT	69	11.5
59	Recurrent neural networks employing Lyapunov exponents for analysis of ECG signals	69	5.31
60	Prediction of cardiac arrest in critically ill patients presenting to the emergency department using a machine learning score incorporating heart rate variability compared with the modified early warning score	68	6.18
61	Comprehensive electrocardiographic diagnosis based on deep learning	67	22.33
62	Improving risk prediction in heart failure using machine learning	67	16.75
63	Artificial Intelligence in Health Care: Bibliometric Analysis	66	22
64	Discrimination power of long-term heart rate variability measures for chronic heart failure detection	65	5.42
65	A Machine Learning System to Improve Heart Failure Patient Assistance	64	7.11

66	Multiple Plasma Biomarkers for Risk Stratification in Patients With Heart Failure and Preserved Ejection Fraction	62	20.67
67	Predicting Survival From Large Echocardiography and Electronic Health Record Datasets Optimization With Machine Learning	62	15.5
68	Predicting all-cause risk of 30-day hospital readmission using artificial neural networks	62	10.33
69	Continuous Wearable Monitoring Analytics Predict Heart Failure Hospitalization The LINK-HF Multicenter Study	60	20
70	Predicting Intensive Care Unit Readmission with Machine Learning Using Electronic Health Record Data	60	12
71	Using EHRs and Machine Learning for Heart Failure Survival Analysis	59	7.38
72	Remote Health Monitoring of Heart Failure With Data Mining via CART Method on HRV Features	59	4.92
73	A review of machine learning techniques in photoplethysmography for the non-invasive cuff-less measurement of blood pressure	58	19.33
74	Predicting readmission risk with institution-specific prediction models	58	7.25
75	PARAMO: A PARAllel predictive MOdeling platform for healthcare analytic research using electronic health records	58	6.44
76	Bispectral analysis and genetic algorithm for congestive heart failure recognition based on heart rate variability	58	5.27
77	Machine learning prediction in cardiovascular diseases: a meta-analysis	57	19
78	Relative Prognostic Importance and Optimal Levels of Risk Factors for Mortality and Cardiovascular Outcomes in Type 1 Diabetes Mellitus	57	14.25

79	Prediction of Heart Disease Using a Combination of Machine Learning and Deep Learning	56	28
80	Diagnosis of Heart Failure With Preserved Ejection Fraction: Machine Learning of Spatiotemporal Variations in Left Ventricular Deformation	56	11.2
81	Predicting the Risk of Heart Failure With EHR Sequential Data Modeling	56	11.2
82	Precision Medicine for Heart Failure with Preserved Ejection Fraction: An Overview	55	9.17
83	An improved support vector machine-based diabetic readmission prediction	54	10.8
84	Machine Learning Algorithm Predicts Cardiac Resynchronization Therapy Outcomes Lessons From the COMPANION Trial	54	10.8
85	Machine learning-based mortality prediction of patients undergoing cardiac resynchronization therapy: the SEMMELWEIS-CRT score	53	17.67
86	Arti ficial Intelligence in Cardiology: Present and Future	53	17.67
87	Machine Learning-Based Three-Dimensional Echocardiographic Quantification of Right Ventricular Size and Function: Validation Against Cardiac Magnetic Resonance	53	13.25
88	Fisher score and Matthews correlation coefficient-based feature subset selection for heart disease diagnosis using support vector machines	53	13.25
89	Identification of novel pheno-groups in heart failure with preserved ejection fraction using machine learning	50	16.67
90	Phenotypic Clustering of Left Ventricular Diastolic Function Parameters Patterns and Prognostic Relevance	50	12.5

91	Regression trees for predicting mortality in patients with cardiovascular disease: What improvement is achieved by using ensemble-based methods?	50	4.55
92	Developing healthcare rule-based expert systems: Case study of a heart failure telemonitoring system	50	4.55
93	Unraveling the Molecular Mechanism of Action of Empagliflozin in Heart Failure With Reduced Ejection Fraction With or Without Diabetes	49	12.25
94	A Multicenter, Scan-Rescan, Human and Machine Learning CMR Study to Test Generalizability and Precision in Imaging Biomarker Analysis	49	12.25
95	Characterising risk of in-hospital mortality following cardiac arrest using machine learning: A retrospective international registry study	49	9.8
96	Investigating the performance improvement of HRV Indices in CHF using feature selection methods based on backward elimination and statistical significance	48	5.33
97	Application of empirical mode decomposition (EMD) for automated identification of congestive heart failure using heart rate signals	47	7.83
98	A mixed-ensemble model for hospital readmission	47	6.71
99	Refining Diagnostic MicroRNA Signatures by Whole-miRNome Kinetic Analysis in Acute Myocardial Infarction	47	4.7
100	Cardiac tissue engineering: state-of-the-art methods and outlook	45	11.25

Table S1 The 100 top-cited papers in heart failure and machine learning.
